# Acceptability among patients of a risk graphic designed to facilitate shared decision making prior to elective abdominal wall hernia repair

**DOI:** 10.1007/s00464-025-12217-y

**Published:** 2025-10-16

**Authors:** Amanda Hernandez, Josh Sinamo, Alex Hallway, Leah Schoel, Ryan Howard, Anne Ehlers, Sean O’Neill, Dana Telem, Michael Rubyan

**Affiliations:** 1 School of Public Health, University of Michigan, Ann Arbor, MI USA; 2 University of Michigan Medical School, 1301 Catherine St, Ann Arbor, MI 48109 USA; 3 Center for Healthcare Outcomes and Policy, University of Michigan, Ann Arbor, MI USA; 4 University of Michigan Institute for Healthcare Policy and Innovation, Ann Arbor, MI USA

**Keywords:** Shared decision-making, Risk management, Elective surgery, Abdominal wall hernia repair

## Abstract

**Background:**

Risk calculators for elective surgery rarely engage patients in designing dynamic visual imagery illustrating how risk can change with preoperative optimization. We conducted a mixed methods study examining the acceptability of illustrated components of a risk calculator for elective hernia repair with patients.

**Methods:**

We conducted semi-structured interviews between August and September 2024. Participants were guided through the visual components of an abdominal wall hernia repair risk calculator and asked to “think aloud” while providing feedback on the tool’s visual appearance. Participants completed a validated scale identifying characteristics for intervention improvement. Quantitative analyses were performed using descriptive statistics, while qualitative data were analyzed using inductive framework analysis.

**Results:**

26 participants completed interviews. Mean age was 49.3 (SD 14.21), mean BMI was 27.8 (SD 6.64). 57.6% identified as female sex, 80.8% identified as White or Caucasian, 11.6% Black or African American, and 3.8% as Hispanic/Latino. 23.1% identified as smokers. Participants agreed that the risk graphic improved their ability to understand risk and behavior change opportunities with a mean agreement of 3.96 (SD 1.31) on a 1–5 agreement scale. Participants noted how clear the graphic was in improving their ability to discuss behavior change opportunities with their surgeon prior to their operation with a mean agreement of 4.04 (SD 1.22) on a 1–5 agreement scale. Qualitatively, we identified four major domains: 1) Delivery Method of the Tool; 2) Usability of the Tool; 3) Interpreting Risk from the Tool; and 4) Value of the Tool. Participants felt the tool sparked conversations with the surgeon, was accessible for shared decision-making, and highlighted the importance of contextualizing risk in decision-making.

**Conclusions:**

Interviews identified areas of improvement for the risk graphic’s visual design and provided signals for how the graphic can be implemented clinically. This study emphasized the importance of incorporating patient perspectives into tools designed for shared decision-making.

**Supplementary Information:**

The online version contains supplementary material available at 10.1007/s00464-025-12217-y.

 Shared decision-making is essential when deciding whether to pursue surgery. Surgeons are ethically obligated to provide clear explanations to their patients about surgical options, approaches, benefits, risks, complications, and alternatives to surgery [1]. These conversations can be complex, lengthy, and overwhelming to patients [2]. Shared decision-making provides a platform for patient’s values and preferences to be discussed and understood by physicians leading to better informed decisions, increased patient satisfaction, and improved treatment adherence. Tools that aid the process and help patients better understand the complex information that they are receiving have been demonstrated to be effective in helping patients actively participate in the decision-making process [3–7].

Shared decision-making tools have been used in vascular surgery, spine surgery, total knee arthroplasty, breast reconstruction, and reduction mammoplasty. These tools include visual aids created to help patients decide between two treatments, [8] educational tools designed to improve patient knowledge [9], and digital decision aids and consultation cards [7, 10–15]. Some tools use web-based calculators and surveys to help patients identify their goals with their surgeon [16–20]. However, they do not incorporate dynamic visual imagery to illustrate risk or risk change. They have been limited in their instructive purpose and primarily focus on educating patients about their options and gauging patient preferences and goals to ameliorate discussion.

While shared decision-making tools are widely used across surgical specialties, patients are rarely involved in designing them, despite evidence that engaging patients in the design process improves their efficacy [21–23]. Furthermore, tools that have been developed with patient input are limited to clinical contexts addressing disabilities, self-management, and self-monitoring rather than surgical intervention [24]. Lack of patient engagement in designing shared decision-making tools is apparent in the setting of abdominal wall hernia repair, one of the most common preference sensitive operations in the U.S. with over 600,000 performed annually [25] – especially for the nearly 25% of patients who undergo this procedure without engaging in preoperative optimization, changing health behaviors prior to an operation, which can reduce complication rates by more than 40%.

In hernia repair, existing shared decision-making tools have focused on using a questionnaire styled tool aimed at improving clinic workflow that does not incorporate a visual aid to guide discussion [26–28]. To address this gap, we created a risk graphic that could be dynamically embedded in a risk calculator for elective abdominal wall hernia repair to help patients make the decision to either pursue or delay their operation. The purpose of this study was to engage patients in examining the acceptability of this risk graphic among patients who may undergo this procedure in the future [13–18].

## Materials and methods

This study used a mixed methods approach to evaluate patient acceptability of the risk graphic combined with the Theoretical Framework of Acceptability (TFA), a validated measure of acceptability to identify attributes of tools that can be improved [29]. The TFA is a validated instrument used to identify ways in which interventions can be improved from the perspective of deliverers and participants across seven constructs (affective attitude, burden, ethicality, intervention coherence, opportunity costs, perceived effectiveness, and self-efficacy) [29].

## Study design

A random sample of participants were drawn from respondents to an online posting on UMHealthResearch, a free and secure tool hosted by the University of Michigan Institute for Clinical and Health Research to facilitate partnerships between health researchers and volunteers in the community. Participants were eligible for the study if they were 18 years of age or older. The target sample size was 30 participants. Participants received a $25 gift card for their time.

## Data collection

**Fig. 1:**
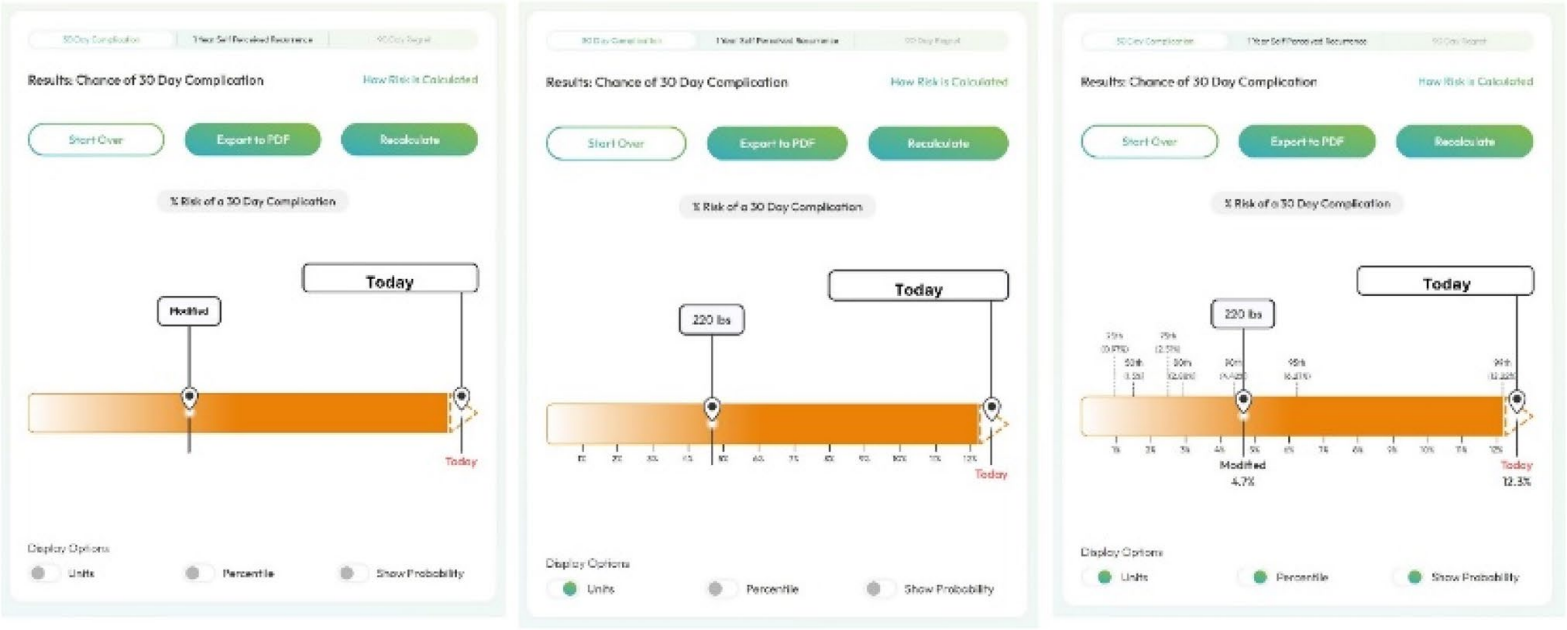
Risk calculator graphic

Four members of the research team (MR, DT, AE, and AH), with experience and training in implementation science and decision science designed the interview guide which was piloted for presentation and clarity (Appendix). This study is reported according to the COREQ (Consolidated Criteria for Reporting Qualitative Research) guidelines [30]. Participants were invited via email to individual sessions facilitated via video conference and informed that researchers leading the study were evaluating the risk graphics. Sessions lasted between 30 and 45 min and took place in August and September of 2024. Each session was conducted by an analyst on the research team (AH) who has expertise in qualitative interviewing. The analyst did not share their assumptions or motivations for facilitating the study with participants. Study procedures were explained after participants logged into the video session and participants were informed that the interview would be video recorded and transcribed. The interviewer used the screen sharing feature in Zoom (San Jose, CA) to display examples of the risk graphics and scenarios in which they would be used (Fig. 1). To understand participant perspectives on acceptability, the concurrent *think-aloud* method [31] was used by asking participants to articulate what they noticed about the risk graphics. No training or contextualization of the graphics were offered before the interview began to avoid biases related to advanced preparation. Interviewers only interjected with contextual information to encourage participants to continue reflecting on their impressions related to the content they were reviewing. Following the interaction with the platform, the interviewer also debriefed each participant individually, using a semi-structured interview guide that assessed overall acceptability and design of the risk graphics and administered the seven-question TFA questionnaire (Appendix) and asked participants to verbally enumerate on how they chose their responses to the questions in the instrument.

Non-participants did not attend the sessions and no relationship with participants was established prior to their participation in the study. No repeated interviews were conducted, and minimal field notes were taken including observations about the most difficult aspects of navigating the platform. Sessions were recorded via Zoom recordings, pseudonymized, and transcribed via Zoom’s automatic transcription feature. Transcripts were not returned to participants for their review. Two study team members independently coded the data and used an inductive framework analysis approach. Data were analyzed in MAXQDA (VERBI Software, Berlin). After 26 interviews were completed, the research team met to discuss data saturation and information power. Since no new domains or sub-themes emerged in later interviews, the team concluded that information power had been achieved, and no additional interviews were required [32]. Major domains were clearly presented in the findings and were illustrated by participant quotations. The study was determined exempt from regulation by the Institutional Review Boards of the University of Michigan Medical School (IRB #HUM00266424).

## Results

### Patient demographics

Twenty-six participants completed interviews. Mean age was 47 years (SD 16.61 years), mean BMI was 27.76 (SD 6.64). 57.6% of participants identified as female sex, 80.8% identified as White or Caucasian, 11.6% Black or African American, and 3.8% as Hispanic or Latino. 23.1% identified as smokers. Table 1 describes the demographics of participants.

**Table 1:** Characteristics of participants who completed interviews, *n* = 26

	No. (%)
Average age (SD)	49.31 (14.21)
*Gender*	
Female	15 (57.6%)
Male	11 (42.3%)
Average BMI (SD)	27.76 (6.64)
*Race*	
White or Caucasian	21 (80.8%)
Black or African American	3 (11.6%)
American Indian or Alaskan Native	1 (3.8%)
Two or more races listed	1 (3.8%)
*Ethnicity*	
Hispanic or Latino	1 (3.8%)
Not Hispanic or Latino	25 (96.2%)
*Smoking Status*	
Both cigarette and other tobacco use	1 (3.8%)
Cigarette tobacco only	2 (7.7%)
Chewing tobacco only	1 (3.8%)
Former tobacco user, no current use	2 (7.7%)
Never smoked	20 (76.9%)
*Current smoking frequency*	
Not at all	23 (88.5%)
Some days	1 (3.8%)
Everyday	2 (7.7%)

## Theoretical framework of acceptability

Results from the TFA questionnaire are reported in Table 2. The mean score for agreement on whether the risk graphic improved participants’ ability to understand risk and behavior change opportunities was 3.96 (SD 1.31) on a 1–5 agreement scale. This was similar to the mean score (4.04; SD 1.22) for agreement on how clear the graphic was for the purposes of improving participants’ ability to discuss behavior change opportunities with their surgeon prior to their operation. The mean score of agreement for whether using the graphic in a risk calculator would interfere with discussion with the surgeon was 1.65 (SD 0.89). and the overall mean score of agreement related to acceptability was 3.81 (SD 1.29).

**Table 2:** Responses to the theoretical framework of acceptability among participants who completed interviews, *n* = 26

	**Mean (Median)**
On a scale of 1 to 5, 1 being very uncomfortable and 5 being very comfortable, how comfortable did you feel using the calculator?	3.24 (1.29)
On a scale of 1 to 5, 1 being no effort at all and 5 being huge effort, how much effort did it take to use the calculator?	2.69 (1.23)
On a scale of 1 to 5, 1 being strongly disagree and 5 being strongly agree, what do you think of the following:	2.92 (1.57)
There are moral or ethical consequences to using the calculator to communicate about risk with my surgeon when discussing undergoing abdominal wall hernia repair	2.92 (1.57)
The calculator has improved my ability to understand risk and behavior change opportunities if I am undergoing abdominal wall hernia repair	3.96 (1.31)
It is clear to me how the calculator will help improve my ability to discuss risk and behavior change opportunities with my surgeon about undergoing abdominal wall hernia repair	4.03 (1.22)
Using the HEROIQ calculator will interfere with my discussion with the surgeon	1.65 (0.89)
On a scale of 1 to 5, 1 being very unconfident and 5 being very confident, how confident do you feel about your ability to understand the calculator?	3.31 (1.46)
On a scale of 1 to 5, 1 being completely unacceptable and 5 being completely acceptable, how acceptable was the calculator to you?	3.81 (1.29)

## Qualitative results

**Fig. 2:**
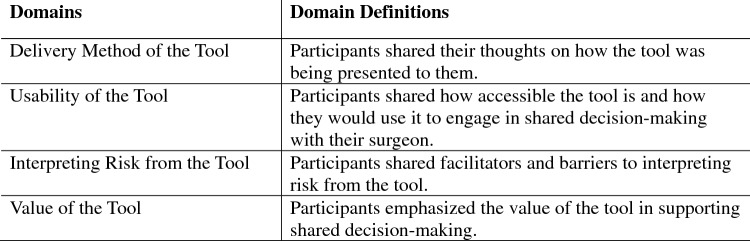
Domains and domain definitions

A total of 645 interview segments were coded. A total of four major domains were identified. Major domains included 1) Delivery Method of the Tool; 2) Usability of the Tool; 3) Interpreting Risk from the Tool; and 4) Value of the Tool. Figure 2 describes the domains and domain definitions, and Table 3 demonstrates illustrative quotes for each domain.

**Table 3:** Illustrative quotes

Domains	Description	Illustrative Quotes
Delivery method of the tool	Using the tool would prepare them for their appointment with the surgeon and provide them with the opportunity to ask more informed questions	*I feel like when I’ve approached, been approached with medical situations in the past like? That’s my 1st question, like, what can I do? And so giving the patient some sense of control (Participant #24)*
		*At least I would be prepared when I met with the doctor, and I could ask him about those potential complications. It would give me time to do a little research, you know, start Googling the procedure on the Internet and finding out about other people’s experiences with that procedure (Participant #3)*
		*It kind of puts me in control of helping things be better. It’s not just all in the surgeons’ hands, I guess. So. It’s something I can do to help lower my risks (Participant #16)*
		*I’d want to discuss whatever the result was with my doctor so I think I think it would be a useful way to help me feel a little more in control over parts of the process (Participant #15)*
	The tool may induce anxiety if they are looking at the tool independently without surgeon input or guidance	*[Using the tool without guidance] would probably just make me way more anxious before I even got in there to hear it (Participant #12)*
		*I probably would do it. But I am prone to getting like worked up and using Google, the Google doctor, and scaring myself about stuff, so I would use with caution, and I might even do something like. Have someone I know do it for me. And then, just like tell me about. Tell me if it’s alarming or not and prefer to like hear it from the surgeon. But, I probably would end up using it. And yeah, I would just put in, I’d be as like honest with it as possible to try and get a sense of it. And I would try to. I would if there were any sources I could look up of like what it’s basing on. I would look those up (Participant #8)*
	Participants discovering their risk prior to the appointment might create an obstacle to pursuing surgery	*That might deter some people from even going to see the doctor like, no, forget it. This is too bad (Participant #16)*
	Participants acknowledged that the time dedicated to these conversations is not sufficient to explain each detail thoroughly and answer any questions	*I would do it just so that I could be well informed to go and then and have a follow up conversation with my surgeon. I would like to be able to ask informed intelligent questions and not waste a whole bunch of time (Participant #21)*
	Participants shared that they would play with a tool that embedded the graphic beforehand to see the risk of their unique situation and see how they can modify their risk	*At the beginning. I will put all my information, but then I will kind of play with it. If it’s something that I can just change because I also feel like I am a patient, and I want the surgery. If I want the surgery. Anyway, I can manipulate my data (Participant #7)*
	Participants might use the tool for insight into which of their characteristics influence their risk	*I think I would definitely do it. And I would. I think I would really appreciate having that tool. And I would definitely find it really enlightening to be able to put in my data and to have a calculated risk before the surgery. I think that would be really cool. I think I might also, let’s say I was given a high risk. I put in my information. I was given a high risk. I might like play with the inputs to see what I could do to lower it. Just because I think that would really worry me if I saw a high risk. So I might want to see, like you know, given that I’m at home, and I’m not sitting with my surgeon asking them what I could do to lower this risk. I might like play with the inputs and see if I could get a lower risk out of curiosity (Participant #1)*
Usability of the tool	Participants who identified themselves as visual learners thought the graphic supplemented their ability to understand	*As I said, I’m a visual person, so it will just enhance our conversation, showing you the visual aid (Participant #4)*
		*I think it’s a really powerful tool. I think giving people a visual representation of their risk is a super smart idea (Participant #1)*
	Participants felt the linear bar graph was easier to interpret	*The single orange bar seems to be simpler and make more sense (Participant #13)*
		*…if you show me this graph [the histogram]. I’m going to say you need to explain this to me because I can’t even start to grasp it (Participant #22)*
	Participants were mixed in their preference of color for the risk bar but felt that orange was more affiliated with risk and danger than blue	*But I think in terms of conveying high risk. I think the orange is better, because that for lack of a better word, that’s almost like a scarier (Participant #1)*
		*Cause, you know, like I said, I feel like oranges signifies like danger, alert caution. You know bad things kind of especially darker orange more concentrated…I feel more at ease looking at the blue (Participant #2)*
Interpreting risk from the tool	Participants were curious about the specific complications and emphasized the importance of contextualizing the quantitative risk in their decision-making process	*I would still need more context. So when we’re talking about complications of the surgery, are we talking about waiting to have the surgery. Are we talking about the surgery? And the risk actually increases after the procedure is done right. I would need a little more clarity there (Participant #21)*
	Participants felt unsettled by the lack of context and specific details of the complications as illustrated by one participant	*Is it deadly? Is it uncomfortable. You know? Is it cosmetic, you know? Is it? You know? Are you gonna die? I don’t know, you know I find it hard to take this seriously. It’s just the doctor would have to, you know. Give me a lot more information to go with this (Participant #11)*
	Participants felt they were making assumptions about what the risk meant	*I was kinda left to draw my own conclusions about what the complications could entail. So maybe I just would have liked more information about, like, you know, what do the things behind these percentages actually represent? Like 12, these 12.3% of people, you know, what do they? Actually what happens to them? (Participant #2)*
	Participants had difficulty understanding the meaning of the self-perceived recurrence complication	*Guess I’d be wanting to know why that is. I’m a little caught up on the risk of me thinking it’s gonna return, even if it hasn’t component (Participant #13)*
	Participants unfamiliar with this complication thought it sounded unreasonable for a hernia repair	*But that sounds like it’s psychological. So why. What would lead me to believe that my wrist, that the bulge had returned. You know, like, why. What? What would lead me? And yeah, is that psychological? Is that normal and is that surgery related like (Participant #13)*
	Higher educational level might be a barrier to interpreting statistical information	*Just because we have some patients that sometimes don’t have a very high education level. Sometimes they can get confused, but I can see I can see that. People with high school education and understanding it well (Participant #7)*
	The quantity of information conveyed by the tool might be a barrier to fully understanding the tool	*It’s too much information. It’s confusing. And I don’t understand the percentiles. So it would probably lead to a longer discussion with the surgeon as to what this is representing (Participant #6)*
	Participants felt talking through the tool might be helpful	*I get the idea of the tool. I don’t like the percentiles. I think that’s confusing. And again, I was just kind of looking at an image. But if there’s like a doctor there, and we’re having a back and forth, then maybe I can understand at best. But if it’s like something that doctors are thinking of, like just giving to patients and like having a patient take it home and like, try to interpret it. It just might be a bit too much. Again. It was just one bar, and it was just complications in general (Participant #16)*
Value of the tool	Participants felt that using the graphic in tandem with a conversation would most strongly impact the participant’s decision	*Well, it it’s helpful. But I would not want to use it. As the only tool to make a decision. It would just give me additional helpful information, but you know, like I I’ve said before, I would need to get more information in order to make an informed decision, and knowing the what percentage of I I’m at of developing a some kind of high risk situation is just additional information, but not enough to base a whole decision for surgery on (Participant #3)*
	The graphic might create a more transparent conversation between the participant and surgeon	*As a surgeon, something to not hide behind, but a way to initiate the discussion instead of (Participant #26)*
	Participants felt more capable of achieving preoperative optimization after reviewing the graphic to identify ways to modify their risk	*My risk today is super high. It’s like off the charts. It’s in the very dark area, my risk could be almost into like a light orangey area, which looks a lot better, you know, more than like a you know, 50% decrease in my risk. It looks like just looking at this graph. That’s probably way safer (Participant #1)*
	The graphic provided participants with optimism that they could optimize their health,	*Makes you feel hopeful that I can get myself into a place where I’m at less risk for surgery (Participant #23)*
	Some participants were motivated by an absolute risk reduction alone	*I mean. Any decrease in risk of complications is worth it to me personally, but that’s a that’s a huge jump, and I would it wouldn’t. I would not even question. I would wait (Participant #4)*
	Participants were curious how intensely the hernia was impeding their quality of life	*So if there was something, if this modification would like, you know, if I’m wearing like a band around my belly, or something to sort of pull the muscle in place or you know a if it wasn’t a big pain like, and I could get by, and it wasn’t a big deal, and it wasn’t was helping with quality of life like I could zip my pants. I could sleep. Everything’s fine. It’s not terribly uncomfortable. I probably would be okay with that like waiting. But I think it would very much depend on, like what that modification was, and like (Participant #24)*
		*So if I’m really in no pain like you said at the beginning, it doesn’t. It’s not causing any pain. It’s not the whatever. The hernia is not causing me any pain. Then it would be worth it to me to start the treatment. Do the treatment, or do the addressing of it, and then have the surgery” (Participant #26)*
		*It probably wouldn’t sway my decision. But if I was at a surgeon’s office and the bulge was bothering me, I think I would have the surgery regardless (Participant #6)*
	Participants felt surgeons supplementing their preoperative conversation with this tool were focused on evidence rather than their subjective opinion	*At least, in my opinion, I think that you’re trying to be as unbiased as you can be. That’s why you’re using like a calculator (Participant #7)*
	The graphic might serve as an objective metric of validation	*Well, for one thing, if that’s what the doctor recommended, I would. You know he’s the expert, so he knows best. And he’s got the. He’s got some paperwork to back up his decision (Participant #3)*

### Domain 1: delivery method of the tool

As part of the first domain, participants expressed that the tool served as a catalyst for conversation with the surgeon and felt more empowered entering the pre-operative appointment. Participants expressed that using the tool prior to their appointment with the surgeon would be beneficial. Some participants emphasized the self-efficacy and autonomy offered using the graphic as it would allow the patient to do further research and develop more nuanced questions and be prepared to discuss the surgery in more detail. By having more agency in the decision-making process, participants could make choices and take steps to directly influence their surgical outcomes rather than passively relying on the surgeon. They also acknowledged that the graphic may make an already time-limited appointment more efficient as it would enhance their confidence around what information to prioritize especially since the time dedicated to these conversations during their preoperative visit would not be sufficient to explain each detail of the graphic thoroughly as well as answer any questions.

Not all participants agreed with this sentiment as some acknowledged that reviewing the graphic may induce anxiety if they are looking at it independently without surgeon input or guidance. They expressed that this might lead them down a path of discovering too much information and entering the preoperative appointment with more suspicions and anxieties than if they reviewed the graphic with their surgeon. They also shared concerns that discovering their risk prior to the appointment might create an obstacle to pursuing surgery and attending the preoperative appointment*.*

### Domain 2: usability of the tool

In the second domain, participants appreciated the opportunity to visualize their risk in tandem with discussion. Participants who identified themselves as visual learners thought the graphic supplemented their ability to understand their risk and behavior change opportunities.

Compared to other visual aids that the interviewer presented, like the histogram or icon array, participants felt that the linear bar graph was simpler and easier to understand. When looking at the histogram or icon array, participants thought they required more sophisticated statistical interpretation. Participants were mixed in their preference of color for the risk bar but felt that orange was more affiliated with risk and danger than blue. Some participants preferred blue either because it was a preferred color for them, or they felt a sense of comfort and relaxation.

### Domain 3: interpreting risk from the tool

In the third domain, participants were curious about the specific complications and emphasized the importance of contextualizing the quantitative risk in their decision-making process. Some participants felt unsettled by the lack of context and specific details of the complications and felt they were making assumptions about what the risk meant for them.

In addition, they had difficulty understanding the meaning of the self-perceived recurrence complication and emphasized the need to explain what each risk was so that they would not be interpreted as unreasonable risks for moving forward with their surgery.

Participants also described barriers to understanding the risk graphic especially related to numeracy. One barrier included difficulty interpreting statistical information such as percentiles leading some participants to believe that a higher educational level might be required to fully understand the tool. Another barrier was the quantity of information conveyed by the tool with added percentiles and statistical details. Participants suggested that if statistical information related to percentiles was included, it might be better to talk through the tool with a surgeon rather than using the tool independently and misinterpreting the results.

### Domain 4: value of the tool

In the fourth domain, participants believed the graphic was successful in facilitating more shared decision-making. They indicated that using the graphic in tandem with a conversation would most strongly impact the participant’s decision on whether to pursue surgery. The graphic might create a more transparent conversation between the participant and surgeon and participants felt they would leave the conversation more fully informed. They felt more capable of achieving preoperative optimization after reviewing the graphic to identify ways to modify their risk. Visualizing a risk reduction from 12.3 to 4.7% evoked sentiments of inspiration as participants noted feeling motivated by an absolute risk reduction alone. This provided participants with optimism that they could optimize their health. In addition, participants felt surgeons supplementing their preoperative conversation with this tool would help them focus on evidence rather than their subjective opinion and that the graphic increased the credibility of the surgeon’s recommendations by serving as an objective metric of validation.

Participants also cautioned that regardless of the presentation of the risk graphic, considerations of their postoperative quality of life would play a key role in their decision to pursue surgery without optimization. This prompted participants to discuss their curiosity related to how painful the hernia was and how intensely the hernia was impeding their quality of life. They indicated that if the pain or symptoms interfered too much, participants might not consider what the risk graphic presents at all*.*

## Discussion

In this study, we elicited key patient feedback on the visual design of a dynamic risk graphic tool for elective abdominal wall hernia repair. There were several key findings. First, participants felt that the tool provided a sense of autonomy and control. Participants preferred a clean and simple linear graph display to demonstrate their risk with consensus to minimize statistical information. In concordance with other shared decision-making tools evaluated by patients, participants felt the tool positively contributed to their ability to contribute to a shared decision-making process with the surgeon.

Participants preferred a clean and simple linear graph display to demonstrate their risk, with an option to include additional statistical detail based on user preference. Participants felt this would help facilitate understanding by avoiding extraneous details for patients who might simply want to know their percent risk of a complication and not the percentile they fall under. Most participants felt the tool improved their ability to discuss risk and expressed that risk discussion should not only include broad statistics and percentages but also information about what each of the complications are and how likely each one is. Participants found that understanding what each complication was helped contextualize their decision and consider their postoperative quality of life in the context of a potential risk. In implementing this graphic with patients, surgeons could incorporate this information during their visit and be sure to ask the patient how much detail regarding each complication is important to them.

Most participants shared that they would use a tool embedding the graphics they reviewed before meeting with their surgeon so they would be more prepared with questions for their surgeon at their appointment. While this sentiment was shared among participants, it is important to note that some participants felt receiving the tool in advance would be discouraging and that reviewing the risk graphic without their surgeon may lead to initial confusion, additional questions, and induce preoperative anxiety.

Based on the feedback and thoughts shared by the participants, there are several ways in which the graphic could be implemented. First, a surgeon or other member of the surgical team could share the risk graphic tool with the patient several days in advance with some background on how the information was collected, what the risk means in terms of what complications are included, and some detail on how to use the tool and toggle on/off statistical information. The surgeon could encourage the patient to review the graphic if they feel comfortable and assure that their preoperative appointment will include discussion about their personal risk for post-operative complications and how they can optimize their health. The simple tool could then be utilized by the patient and easily manipulated for their interests with the option to include as much or as little statistical information they feel comfortable with. At the appointment, the surgeon could initiate the discussion with the patient and discover if the patient used the tool and what they understood. From there, the surgeon and patient could engage in shared decision-making while using the risk graphic together to identify the patient’s preferences and answer the patient’s questions about the surgical intervention. If effective, the patient and surgeon would feel informed, prepared, and confident about the decision for surgery moving forward. In contrast, the surgeon could present the graphic to the patient and explain how risks might be modified through preoperative optimization. Future research could explore the efficacy of these different approaches in disseminating and implementing the risk graphic as part of a shared decision-making tool.

This study is not without limitations. First, participants were recruited from a single academic medical center limiting the generalizability of the perspectives of participants receiving elective hernia repair. However, participants identified as living in many different geographical areas in Southeast Michigan. Second, participants were not required to have had prior surgeries of any kind. This might have made it challenging for them to understand the complexities of a risk graphic as their perceptions could be based on theoretical knowledge rather than experiences with medical procedures or risk management. However, evaluating risk graphics with participants who have not undergone the procedure is consistent with previously published literature [21]. We also did not exclude participants who had already undergone a hernia repair. As a result, participants with prior experience may have a different understanding of risk, expectations, and outcomes compared to those considering the surgery for the first time. Their previous experiences with hernia repair could influence how they interpret the risk graphic. Additionally, participants might base their feedback more on their personal emotional reaction to surgery in general rather than the graphic’s usefulness or accuracy limiting our ability to draw conclusions from their perspectives.

## Conclusion

Our findings identify key strengths and weaknesses of the visual design of the risk graphics that could be embedded in a risk calculator based on patient perspectives. Overall, participants found the risk graphic to be both acceptable and useful. However, for clinical implementation, additional studies will need to examine whether patients can use the tool to prepare for their visit or if surgeons should be prepared to discuss the tool with patients without sharing it with patients prior to the discussion. This insight provides opportunities to refine its design and improve usability and implementation with patients. The study emphasizes the importance of incorporating patient perspectives into the design of a tool that will ultimately be used in facilitating shared decision-making for preference sensitive operations.

## Supplementary Information

Below is the link to the electronic supplementary material.

Supplementary file1 (DOCX 26 KB)

## References

[1] Boss EF, Mehta N, Nagarajan N et al (2016) Shared decision making and choice for elective surgical care: a systematic review. Otolaryngol Head Neck Surg 154(3):405–420. 10.1177/0194599815620558 26645531 10.1177/0194599815620558 PMC4857133

[2] Caverly TJ, Hayward RA (2020) Dealing with the lack of time for detailed shared decision-making in primary care: everyday shared decision-making. J Gen Intern Med 35(10):3045–3049. 10.1007/s11606-020-06043-2 32779137 10.1007/s11606-020-06043-2 PMC7572954

[3] Yen RW, Durand MA, Harris C et al (2020) Text-only and picture conversation aids both supported shared decision making for breast cancer surgery: analysis from a cluster randomized trial. Patient Educ Couns 103(11):2235–2243. 10.1016/j.pec.2020.07.015 32782181 10.1016/j.pec.2020.07.015

[4] Politi MC, Lee CN, Philpott-Streiff SE, Foraker RE, Olsen MA, Merrill C, Tao Y, Myckatyn TM (2020) A randomized controlled trial evaluating the BREASTChoice tool for personalized decision support about breast reconstruction after mastectomy. Ann Surg. 271(2):230–237. 10.1097/SLA.0000000000003444 31305282 10.1097/SLA.0000000000003444

[5] de Mik SML, Stubenrouch FE, Balm R, Ubbink DT (2021) Development of three different decision support tools to support shared decision-making in vascular surgery. Patient Educ Couns 104(2):282–289. 10.1016/j.pec.2020.11.036 33277102 10.1016/j.pec.2020.11.036

[6] Sepucha K, Atlas SJ, Chang Y et al (2017) Patient decision aids improve decision quality and patient experience and reduce surgical rates in routine orthopaedic care. J Bone Joint Surg 99(15):1253–1260. 10.2106/JBJS.16.01045 28763411 10.2106/JBJS.16.01045

[7] Bozic KJ, Belkora J, Chan V et al (2013) Shared decision making in patients with osteoarthritis of the hip and knee. J Bone Joint Surg 95(18):1633–1639. 10.2106/JBJS.M.00004 24048550 10.2106/JBJS.M.00004

[8] Yen RW, Durand MA, Harris C et al (2020) Text-only and picture conversation aids both supported shared decision making for breast cancer surgery: analysis from a cluster randomized trial. Patient Educ Couns 103(11):2235–2243. 10.1016/j.pec.2020.07.015 32782181 10.1016/j.pec.2020.07.015

[9] Politi MC, Lee CN, Philpott-Streiff SE et al (2020) A randomized controlled trial evaluating the BREASTChoice tool for personalized decision support about breast reconstruction after mastectomy. Ann Surg 271(2):230–237. 10.1097/SLA.0000000000003444 31305282 10.1097/SLA.0000000000003444

[10] Stubenrouch FE, Peters LJ, de Mik SML et al (2022) Improving shared decision making in vascular surgery: a stepped wedge cluster randomised trial. Eur J Vasc Endovasc Surg 64(1):73–81. 10.1016/j.ejvs.2022.04.016 35483576 10.1016/j.ejvs.2022.04.016

[11] Ifelayo OI, Brito JP, Hargraves IG, Larson AN (2021) Development of a shared decision-making tool for adolescents with scoliosis to decide between observation versus fusion surgery. J Pediatr Orthop 41(Suppl 1):S70–S74. 10.1097/BPO.0000000000001800 34096541 10.1097/BPO.0000000000001800

[12] Mertz K, Shah RF, Eppler SL et al (2020) A simple goal elicitation tool improves shared decision making in outpatient orthopedic surgery: a randomized controlled trial. Med Decis Making 40(6):766–773. 10.1177/0272989X20943520 32744134 10.1177/0272989X20943520 PMC7647048

[13] Vaisson G, Provencher T, Dugas M et al (2021) User involvement in the design and development of patient decision aids and other personal health tools: a systematic review. Med Decis Making 41(3):261–274. 10.1177/0272989X20984134 33655791 10.1177/0272989X20984134

[14] Tierney WM, Henning JM, Altillo BS et al (2023) User-centered design of a clinical tool for shared decision-making about diet in primary care. J Gen Intern Med 38(3):715–726. 10.1007/s11606-022-07804-x 36127543 10.1007/s11606-022-07804-x PMC9971535

[15] de Mik SM, Stubenrouch FE, Legemate DA, Balm R, Ubbink DT (2020) Improving shared decision-making in vascular surgery by implementing decision support tools: study protocol for the stepped-wedge cluster-randomised OVIDIUS trial. BMC Med Inform Decis Mak 20(1):172. 10.1186/s12911-020-01186-y 32703205 10.1186/s12911-020-01186-y PMC7376920

[16] Stubenrouch FE, Peters LJ, de Mik SML et al (2022) Improving shared decision making in vascular surgery: a stepped wedge cluster randomised trial. Eur J Vasc Endovasc Surg 64(1):73–81. 10.1016/j.ejvs.2022.04.016 35483576 10.1016/j.ejvs.2022.04.016

[17] Bhat S, Wang AT, Wood F, Orgill DP (2022) Visual preoperative risk depiction tools for shared decision-making: a pilot study from the surgeon’s perspective. Plast Reconstr Surg 10(11):e4690. 10.1097/GOX.0000000000004690 10.1097/GOX.0000000000004690 PMC9708169 36467117

[18] Moulton H, Tosteson TD, Zhao W, Pearson L, Mycek K, Scherer E, Weinstein JN, Pearson A, Abdu W, Schwarz S, Kelly M (2018) Considering spine surgery: a web-based calculator for communicating estimates of personalized treatment outcomes. Spine. 43(24):1731–1738. 10.1097/BRS.0000000000002723 29877995 10.1097/BRS.0000000000002723 PMC6279474

[19] Ifelayo OI, Brito JP, Hargraves IG, Larson AN (2021) Development of a shared decision-making tool for adolescents with scoliosis to decide between observation versus fusion surgery. J Pediatric Orthop 41:S70–S74. 10.1097/BPO.0000000000001800 10.1097/BPO.0000000000001800 34096541

[20] Mertz K, Shah RF, Eppler SL, Yao J, Safran M, Palanca A, Hu SS, Gardner M, Amanatullah DF, Kamal RN (2020) A simple goal elicitation tool improves shared decision making in outpatient orthopedic surgery: a randomized controlled trial. Med Decis Mak 40(6):766–73. 10.1177/0272989X20943520 10.1177/0272989X20943520 PMC7647048 32744134

[21] Vaisson G, Provencher T, Dugas M et al (2021) User involvement in the design and development of patient decision aids and other personal health tools: a systematic review. Med Decis Making 41(3):261–274. 10.1177/0272989X20984134 33655791 10.1177/0272989X20984134

[22] Tierney WM, Henning JM, Altillo BS et al (2023) User-centered design of a clinical tool for shared decision-making about diet in primary care. J Gen Intern Med 38(3):715–726. 10.1007/s11606-022-07804-x 36127543 10.1007/s11606-022-07804-x PMC9971535

[23] Sañudo Y, Akoglu C, Rietjens JAC, Snelders D, Stiggelbout AM, Sierra-Pérez J (2025) The implementation of design methodologies for supporting shared decision making in healthcare services: a systematic review. Patient Educ Couns 131:108551. 10.1016/j.pec.2024.108551 39577307 10.1016/j.pec.2024.108551

[24] Vaisson G, Provencher T, Dugas M et al (2021) User involvement in the design and development of patient decision aids and other personal health tools: a systematic review. Med Decis Making 41(3):261–274. 10.1177/0272989X20984134 33655791 10.1177/0272989X20984134

[25] Schlosser KA, Renshaw SM, Tamer RM, Strassels SA, Poulose BK (2023) Ventral hernia repair: an increasing burden affecting abdominal core health. Hernia 27(2):415–421. 10.1007/s10029-022-02707-6 36571666 10.1007/s10029-022-02707-6

[26] Casson CA, Kushner BS, Holden TR, Majumder A, Blatnik JA, Holden SE (2025) Patient expectations and decisional regret in the management of ventral hernias. Surg Endosc 39(1):522–529. 10.1007/s00464-024-11318-4 39414667 10.1007/s00464-024-11318-4

[27] Gleason F, Feng K, Herbey I, Shorten A, Chu DI, Parmar AD (2021) Patient, nurse, medical assistant, and surgeon perspectives inform the development of a decision support tool for inguinal hernia surgery: a qualitative analysis. Am J Surg 222(2):272–280. 10.1016/j.amjsurg.2021.01.009 33514451 10.1016/j.amjsurg.2021.01.009

[28] Latenstein CSS, van Wely BJ, Klerkx M, Meinders MJ, Thomeer B, de Reuver PR (2019) Reduced elective operation rates and high patient satisfaction after the implementation of decision aids in patients with gallstones or an inguinal hernia. World J Surg 43(9):2149–2156. 10.1007/s00268-019-05007-w 31011818 10.1007/s00268-019-05007-w

[29] Atkins L, Francis J, Islam R et al (2017) A guide to using the theoretical domains framework of behaviour change to investigate implementation problems. Implement Sci 12(1):77. 10.1186/s13012-017-0605-9 28637486 10.1186/s13012-017-0605-9 PMC5480145

[30] Tong A, Sainsbury P, Craig J (2007) Consolidated criteria for reporting qualitative research (COREQ): a 32-item checklist for interviews and focus groups. Int J Qual Health Care 19(6):349–357. 10.1093/intqhc/mzm042 17872937 10.1093/intqhc/mzm042

[31] Jaspers M, Steen T, Bos C, Geenen M (2004) The think aloud method: a guide to user interface design. Int J Med Inform 73(11–12):781–795. 10.1016/j.ijmedinf.2004.08.003 15491929 10.1016/j.ijmedinf.2004.08.003

[32] Malterud K, Siersma VD, Guassora AD (2015) Sample size in qualitative interview studies: guided by information power. Qual Health Res 26(13):1753–1760. 10.1177/1049732315617444 10.1177/1049732315617444 26613970

