# Supplementary material for: Acceptability among patients of a risk graphic designed to facilitate shared decision making prior to elective abdominal wall hernia repair
Source: Surg Endosc. 2025 Oct 16;40(1):598–608. doi: 10.1007/s00464-025-12217-y (PMC12823723; doi:10.1007/s00464-025-12217-y)
Supplement: Supplementary file 1 — Supplementary file1 (DOCX 26 KB) [file 464_2025_12217_MOESM1_ESM.docx]

# **Appendix. Interview Guide**

**PART 1**

**SCENARIO 1**

This is what I show you:

*Show risk graphic with one marker on* ***left hand side*** *(Scenario 1)*

1. *Markers only (Slide 1)*
2. *Markers + units (Slide 2)*
3. *Markers + units + probability (Slide 3)*
4. *Markers + units + probability + distribution (Slide 4)*

*FOR EACH SLIDE ASK THE FOLLOWING:*

- How does this make you feel?
- What does this look like to you?
- What do you think about this?
- Would you have surgery? Why or why not? Tell us what you are thinking about.

**SCENARIO 2**

Now, what if I showed you this?

*Show risk graphic with one marker on* ***right hand side*** *(Scenario 2)*

1. *Markers only (Slide 5)*
2. *Markers + units (Slide 6)*
3. *Markers + units + probability (Slide 7)*
4. *Markers + units + probability + distribution (Slide 8)*

*FOR EACH SLIDE ASK THE FOLLOWING:*

- How does this make you feel?
- What does this look like to you?
- What do you think about this?
- Would you have surgery? Why or why not? Tell us what you are thinking about.

**SCENARIO 3**

What if you had a condition that would take many months to a year to address, but if you addressed it, I show you this:

*Show risk graphic with the marker for* ***right hand side*** *and a marker in the* ***middle section*** *of the graphic (Scenario3)*

1. *Markers only (Slide 9)*
2. *Markers + units (Slide 10)*
3. *Markers + units + probability (Slide 11)*
4. *Markers + units + probability + distribution (Slide 12)*

*FOR EACH SLIDE ASK THE FOLLOWING:*

- How does this make you feel?
- What does this look like to you?
- What do you think about this?
- Would you have surgery? Why or why not? Tell us what you are thinking about.

**PART 2**

**Results**

- What do you think about these results?
  - Would the way this is displayed change your decision?
- How would you respond if your surgeon used the results of this tool to encourage you to improve your health before surgery?
- If this calculator and your surgeon told you your **risk of a complication after surgery** was higher than most other people’s risk, what questions would you have for your surgeon?
- If this calculator and your surgeon told you that **the risk of you believing that the bulge has returned (even if it has not)** was higher than most other people’s risk, what questions would you have for your surgeon?

What do you think about this tool in general?

**Other Display Options**

- What if I showed you your risk on a graphic like this one (*Slide 13 – Histogram*)
  - Would you prefer this graphic or the bar we looked at in the others?
- What if I showed you your risk on a graphic like this one (*Slide 14 – Icon Array*)
  - Would you prefer this graphic or the bar we looked at in the others?
- What if I showed you a blue colored bar instead of the orange bar (*Slide 15 – Blue Bar*)
  - Would you prefer this blue bar or the orange bar we looked at in the others?

**PART 3**

**Acceptability of the Tool** *(for each question below, read the question and the scale, record the response on the scale and follow up with questions about why the participant chose that response (i.e. tell me more about that).  Be sure to prompt participant to provide a numeric response (whole numbers only) for each scale if they do not.*

How comfortable did you feel using the calculator? (TFA Acceptability Questionnaire, Affective attitude)

| **Very uncomfortable** | **Uncomfortable** | **No Opinion** | **Comfortable** | **Very Comfortable** |
| --- | --- | --- | --- | --- |
| 1 | 2 | 3 | 4 | 5 |

How much effort did it take to use the calculator? (TFA Acceptability Questionnaire, Burden)

| **No effort at all** | **A little effort** | **No Opinion** | **A lot of effort** | **Huge effort** |
| --- | --- | --- | --- | --- |
| 1 | 2 | 3 | 4 | 5 |

There are moral or ethical consequences to using the calculator to communicate about risk with my surgeon when discussing undergoing abdominal wall hernia repair (TFA Acceptability Questionnaire, Ethicality)

| **Strongly Disagree** | **Disagree** | **No Opinion** | **Agree** | **Strongly Agree** |
| --- | --- | --- | --- | --- |
| 1 | 2 | 3 | 4 | 5 |

The calculator has improved my ability to understand risk and behavior change opportunities if I am undergoing abdominal wall hernia repair. (TFA Acceptability Questionnaire, Perceived effectiveness)

| **Strongly Disagree** | **Disagree** | **No Opinion** | **Agree** | **Strongly Agree** |
| --- | --- | --- | --- | --- |
| 1 | 2 | 3 | 4 | 5 |

It is clear to me how the calculator will help improve my ability to discuss risk and behavior change opportunities with my surgeon about undergoing abdominal wall hernia repair. (TFA Acceptability Questionnaire, Intervention Coherence)

| **Strongly Disagree** | **Disagree** | **No Opinion** | **Agree** | **Strongly Agree** |
| --- | --- | --- | --- | --- |
| 1 | 2 | 3 | 4 | 5 |

How confident did you feel about your ability to understand the calculator? (TFA Acceptability Questionnaire, Self-efficacy)

| **Very Unconfident** | **Unconfident** | **No Opinion** | **Confident** | **Very Confident** |
| --- | --- | --- | --- | --- |
| 1 | 2 | 3 | 4 | 5 |

Using the HEROIQ calculator will interfere with my discussion with the surgeon. (TFA Acceptability Questionnaire, Opportunity costs)

| **Strongly Disagree** | **Disagree** | **No Opinion** | **Agree** | **Strongly Agree** |
| --- | --- | --- | --- | --- |
| 1 | 2 | 3 | 4 | 5 |

How acceptable was the calculator to you? (TFA Acceptability Questionnaire, General Acceptability)

| **Completely Unacceptable** | **Unacceptable** | **No Opinion** | **Acceptable** | **Completely Acceptable** |
| --- | --- | --- | --- | --- |
| 1 | 2 | 3 | 4 | 5 |
